# Can anodised zirconium implants stimulate bone formation? Preliminary study in rat model

**DOI:** 10.1007/s40204-014-0024-9

**Published:** 2014-06-11

**Authors:** Maria R. Katunar, Andrea Gomez Sanchez, Josefina Ballarre, Matias Baca, Carlos Vottola, Juan C. Orellano, Hanna Schell, Gustavo Duffo, Silvia Cere

**Affiliations:** 1 Corrosion Division, INTEMA, Universidad Nacional de Mar del Plata-CONICET, Juan B. Justo 4302, B7608FDQ Mar del Plata, Argentina; 2 Traumatologia y Ortopedia, Hospital Interzonal General de Agudos “Oscar Alende”, Mar del Plata, Argentina; 3 Center of Muskuloeskeletal Surgery, Charite-Universitätsmedizin Berlin, Augustenburger Plats 1, D-13353 Berlin, Germany; 4 Departamento de Materiales, Comisión Nacional de Energía Atómica, CONICET, Av. Gral. Paz 1499, B1650KNA San Martín, Buenos Aires Argentina; 5 Universidad Nacional de Gral. San Martín, Av. Gral. Paz 1499, B1650KNA San Martín, Buenos Aires Argentina

**Keywords:** Orthopaedic implant, Osseointegration, Zirconium, Anodisation, In vivo model

## Abstract

The mechanical properties and good biocompatibility of zirconium and some of its alloys make these materials good candidates for biomedical applications. The attractive in vivo performance of zirconium is mainly due to the presence of a protective oxide layer. In this preliminary study, the surface of pure zirconium modified by anodisation in acidic media at low potentials to enhance its barrier protection given by the oxides and osseointegration. Bare, commercially pure zirconium cylinders were compared to samples anodised at 30 V through electrochemical tests and scanning electron microscopy (SEM). For both conditions, in vivo tests were performed in a rat tibial osteotomy model. The histological features and fluorochrome-labelling changes of newly bone formed around the implants were evaluated on the non-decalcified sections 63 days after surgery. Electrochemical tests and SEM images show that the anodisation treatment increases the barrier effect over the material and the in vivo tests show continuous newly formed bone around the implant with a different amount of osteocytes in their lacunae depending on the region. There was no significant change in bone thickness around either kind of implant but the anodised samples had a significantly higher mineral apposition, suggesting that the anodisation treatment stimulates and assists the osseointegration process. We conclude that anodisation treatment at 30 V can stimulate the implant fixation in a rat model, making zirconium a strong candidate material for permanent implants.

## Introduction

In dental and orthopaedic implants, metals are used when mechanical stability and resistance to cyclic loading are needed. Several materials and surface modification treatments have been developed to provide specific properties such as degradation and corrosion protection, improved tissue integration, and controlled friction at interfaces (Mendonça et al. 2008; Thomsen and Gretzer 2001; Ellingsen 2000; Ballarre et al. 2002). Surface modification effects have been examined experimentally in vivo and in vitro (Gu et al. 2004; Kim et al. 2004; Lee et al. 2004).

It is well known that the clinical success of orthopaedic implants depends on two main factors: initial fixation due to osseointegration in the first few months; and maintenance of the fixation over the long term (Bonsignore et al. 2013). Branemark et al. (1959, 1983) defined the term osseointegration as the “direct structural and functional connection between ordered, living bone and the surface of a load-carrying implant”. Since then, this concept has been redefined at multiple levels such as clinically (Adell et al. 1981), anatomically (Branemark 1983), histologically and ultrastructurally (Linder et al. 1983). Currently, an implant is considered as “osseointegrated” when there is no progressive relative movement between the implant and the bone in direct contact.

Cementless prosthesis have been suggested to have the minimal stress shielding and even superior survival rate (Moreland and Moreno 2001; Emerson et al. 2002; Yamada et al. 2009) which make them the primary choice for young patients. Great effort is put into the development of new implants that are designed for better cementless fixation. A variety of surface modifications have been studied and applied to implants to achieve long-term fixation to the host bone by osseointegration (Ergun et al. 2003; Karthega et al. 2010; Ballarre et al. 2009, 2010; Lind et al. 1999).

As a valve metal, zirconium is covered with a thin film of “native” ZrO_2_ oxide in air mainly amorphous and homogeneous that acts as a protective layer from corrosion in a wide range of media due to their low electronic conductivity and thermodynamic stability, which in turn is biocompatible (Sollazzo et al. 2008). The properties of the oxide layer may be the reasons for the biocompatibility leading to favourable tissue responses to Ti and Zr in comparison to many other metal implants, since primary interaction at the implant–tissue interface is dependent on the surface properties of the oxide layer and not on the bulk material (Sul 2002; Gomez Sanchez et al. 2011). The physicochemical and electrochemical properties of the oxide film and its long-term stability in biological environments play a key role for the biocompatibility of metallic implants. It has been demonstrated that an artificial increase of the thickness and changes in the topography of the native oxide will result in very strong reinforcement of the bone response (Albrektsson et al. 2000; Sul et al. 2002). Further thickening of the surface oxide layer may be performed by different routes, including thermal treatments in air (Benaboud et al. 2007), immersion in peroxide (Pan et al. 1994) or anodization (Preusser et al. 1994; Pauporté and Finne 2006). Zirconium (Zr) and its alloys have been studied for being used in the nuclear power industry and have been recently commercialized for its use in medical implants, especially for total knee and hip replacements after hydrothermally grown oxide (Tsutsumi et al. 2010). Zr and Zr alloys have greater strength, lower cytotoxicity and lower magnetic susceptibility than Titanium (Ti) (Yamamoto et al. 1998). These advantageous properties make Zr and its alloys good promising candidates as materials in orthopaedic surgery. In order to enhance osseointegration of endosseous Zr implants, modification of the surface is needed with the aim of improving the capability for calcium phosphate formation and osteoblast adhesion and proliferation (Wang and Luo 2012). As surface properties can influence the bone formation and healing around the implant, it is of great importance to evaluate long-term response of tissues. Since an additional requirement of metal implants is the corrosion resistance in body fluids for long periods, the electrochemical in vitro response of anodized zirconium was systematically studied to determine the effect of the surface modification process on the corrosion resistance of this metal (Thomsen and Gretzer 2001; Lee et al. 2004). It has been demonstrated that the chemistry and the topography of the surface oxide formed by Zr anodization in phosphoric acid are simultaneously modified (Gomez Sanchez et al. 2011). The oxide formed on the surface is mainly monoclinic ZrO_2_ with the incorporation of phosphates from the electrolyte, and no dissolution of the oxide occurs in the electrolytic media, increasing the corrosion resistance of the anodized samples when compared with the untreated ones. Surface modification of zirconium by anodization has proved to be a treatment that can keep corrosion parameters in low values in simulated body fluid while being able to promote Ca–P compounds’ precipitation on the surface endorsing the bioactivity of the material and promoting bone regeneration in vitro (Gomez Sanchez et al. 2013). The preliminary in vivo test demonstrated that although no Ca–P compounds were detected in the Zr with the native oxide, new bone was developed as in the anodized Zr, Although osseointegration was observed for anodized and non-anodized samples, the quality, kinetics of growing and maturity are not well established (Gomez Sanchez et al. 2013; Hoerth et al. 2014). Since the primary interaction between the tissue and the implant in through the surface, the understanding of their interaction for long-term service deserves further research. The aim of this study is to correlate the surface modification of anodised zirconium and its in vitro behaviour in corrosion studies with the osseointegration response in vivo, employing a histological approach.

## Materials and methods

### Implants and surface treatment

Commercially pure zirconium cylinders (99.5 % Roberto Cordes SA, Argentina) of 40–50 mm length and 1-mm diameter were used. Two surface conditions were compared: as received pure zirconium (control) and zirconium anodised at a constant potential of 30 V during 60 min in 1 mol L^−1^ H_3_PO_4_ (anodised). The sample conditioning and oxide growth details have been previously reported (Gomez Sanchez et al. 2013).

The overall surface morphology of the specimens was observed by scanning electron microscopy (SEM) (JEOL JSM-6460LV, Japan) at 15 kV.

### Electrochemical studies in simulated body fluid solution (SBF)

In order to evaluate corrosion resistance of the materials in service, electrochemical assays were conducted in SBF. SBF solution has an ion concentration similar to that of blood plasma, and it has been extensively used to evaluate the in vitro behaviour of biomaterials (Kokubo and Takadama 2006; Kokubo et al. 2009). All reagents were provided by Sigma–Aldrich (analytical grade, 85.0 %), and deionised water (18.2 MΩ cm, Millipore) was used throughout. The solution was buffered to pH 7.4 with concentrated HCl and tris(hydroxymethyl)aminomethane (tris, 0.05 mol L^−1^).

Control and anodised electrodes were electrochemically studied in SBF using a conventional three electrode cell with a saturated calomel electrode (SCE, Radiometer Analytical, France) as reference and a platinum wire as counter electrode. Measurements were performed after 24 h of immersion in SBF. Before each measurement, the potential was left to stabilize for 40 min at open circuit potential. A Reference 600TM Potentiostat–Galvanostat-ZRA (Gamry Instruments, USA) was used.

Potentiodynamic polarization curves were performed from the open circuit potential to 1.5 V (or until the current density reached a value of 10^−2^ A cm^−2^) and back at a sweep rate of 0.002 Vs^−1^.

Electrochemical impedance spectroscopy (EIS) measurements were carried out using a PCI4 750/potentiostat/galvanostat/ZRATM (Gamry Instruments, USA). The amplitude of the perturbation signal was 10 mV rms, and the impedance was measured between 10^−2^ and 10^6^ Hz.

### Animal model and surgery

#### Animals

All the experiments were approved by the Bioethics Committee HIEMI-HIGA, (Mar del Plata, October 2011). Twelve-week-old male WKAH/Hok rats (*n* = 6) weighting 300–330 g were used in this study. The animals were divided in two groups for each type of surface treatment: control and anodised. All animals were housed in a temperature controlled room with a 12-h alternating light–dark cycle and were given water and food ad libitum throughout the study.

#### Surgical procedure

**Fig. 1:**
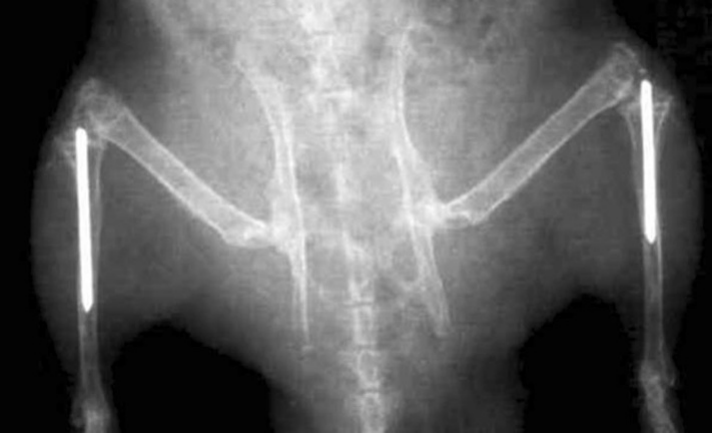
Radiography or a rat tibia including implant

Rats were anaesthetized with fentanyl citrate and droperidol (Janssen-Cilag Lab, Johnson and Johnson, Madrid, Spain) according to their weight, and the surgery region was cleaned with antiseptic soap. The animals were placed in a supine position, and the implantation site was exposed through the superior part of the tibia’s internal face. A region of around 0.5-cm diameter was scraped in the tibia and femur plateau, and a hole was drilled using a hand drill at low speed with a 0.15-cm diameter burr. The implantation site was irrigated with physiological saline solution during the drilling procedure for cleaning and cooling proposes. The control and anodised implants were placed by press-fit into tibia extending into the medullar canal. Conventional X-ray radiographs were taken after surgery for control purposes (Fig. 1).

### Bone labelling with fluorochromes

**Fig. 2:**
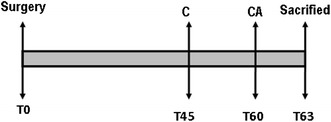
Time line showing the fluorochrome-labelling protocol

Fluorescent dyes were used to follow dynamic calcium deposition over time (Fig. 2). Polyfluorochrome tracers, calcein green (30 mg/kg, C0875 SIGMA) and alizarin complexone (30 mg/kg, A3882 SIGMA) were injected intraperitoneally. They bind to calcium ions and later can be incorporated at mineralization sites in the form of hydroxyapatite crystals. The florescent label demarcates the mineralization front at the time of administration and can be detected in histological sections without any further staining or decalcification (Van Gaalen et al. 2010; Rahn 2003). The rats were injected with calcein green 45 days after implantation and then with alizarin complexone 60 days after implantation. The animals were killed 63 days after implantation. The fluorescent dyes can be detected in histological sections with a fluorescent microscope (Nikon Eclipse Ti, Tokyo, Japan) with appropriate filters giving an indication of new matrix deposition over time (Kajiwara et al. 2005).

### Histological analysis

#### Tissue processing and sampling

Sixty-three days after implantation, six rats with control and anodised implants (all individuals from different litters) were deeply anaesthetized with Ketamine/Xylazine (75, 10 mg/kg). They were perfused through the cardiac left ventricle, initially with 15 mL of a cold saline solution containing 0.05 % w/v NaNO_2_ plus 50 IU of heparin and subsequently with 150 mL of a cold fixative solution containing 4 % paraformaldehyde in 0.1 mol L^−1^ phosphate buffer, pH 7.4. The retrieved samples were cleaned from surrounding soft tissues and fixed in neutral 10 wt% formaldehyde for 24 h. Then they were dehydrated in a series of alcohol–water mixtures followed by a methacrylated solution, and finally embedded in a polymethyl methacrylate (PMMA) solution and polymerized. The PMMA embedded blocks were cut with a low-speed diamond blade saw (Buehler GmbH) cooled with water. Sections were made 100 µm thick for dynamic histomorphometry assays.

#### Histological observations

Proximal tibia cross sections transversal to the central long axis of the tibia (*n* = 3 p/surface treatment group) were prepared and cut as described above. Three sections from the proximal region were selected. Toluidine staining allows observation and identification/discrimination of tissues (e.g. bone and bone marrow) around the metal–implant interface without removing the PMMA before the histological examination in a light microscope (Nikon Eclipse Ti, Tokyo, Japan).

Histomorphometric analysis of the newly bone thickness of newly formed bone in contact with the implant was evaluated using the software ImageJ 1 (open source: http://rsb.info.nih.gov/ij/features.html). The area of the primary press-fit contact of the implant and bone was excluded from the analysis. Thus, only new bone was analysed in the study (Miettinen et al. 2009).

#### Mineral apposition rate (MAR)

The extent of newly formed bone around the implant was measured from 200× fluorescence microscopy images for each type of implant. At 45 and 60 days after implantation, the distances from the bone surface facing the implant to the calcein-labelled line and alizarine complexone-labelled line were measured to evaluate the amount of newly formed bone. The nomenclature and symbols used in conventional bone histomorphometry are those describe by Parfitt et al. (1987). The mineral apposition rate (MAR, µm/day) is the rate at which mineral accretion occurs at a remodelling site during the period of bone formation. MAR is a fundamental histomorphometric variable, and it is a reliable measure of osteoblast function.

### Statistical analysis

In this study, the data are shown in the form of mean value ± σ_mean_ (standard deviation of the mean). Differences between the groups were assessed by a non-parametric test. Mann–Whitney was performed using GraphPad In Stat version 3.00 (Graph Pad Software). All statistical analysis was considered significant when *p* value <0.05.

## Results

### Surface analysis

**Fig. 3:**
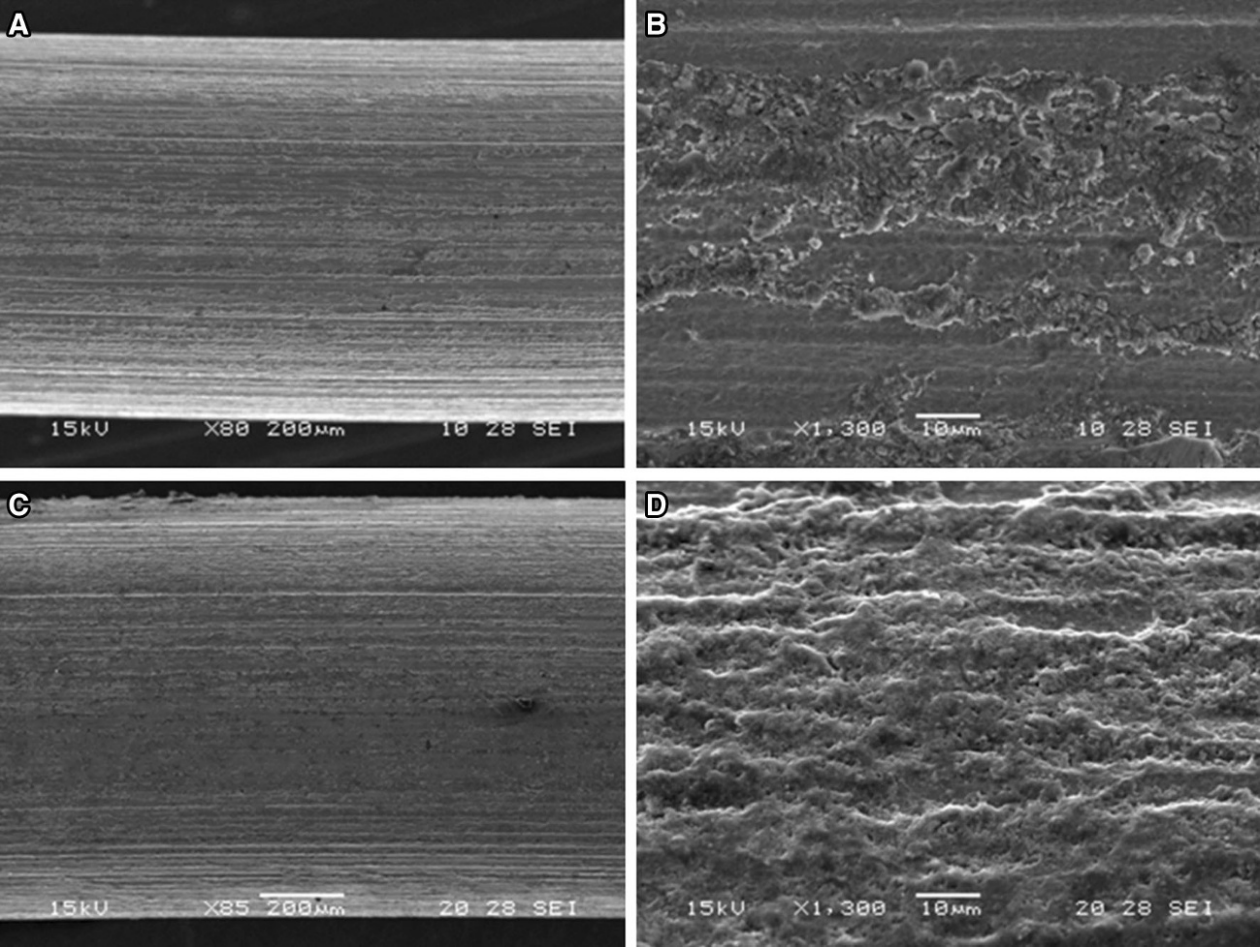
SEM micrographs of zirconium cylinders with different magnification. **a** and **b** control, **c** and **d** anodised

Figure 3 presents the SEM micrographs of zirconium samples (control and anodised). As expected, no major differences are visible between both surface conditions of zirconium. Parallel lines along the length of the samples correspond to the marks of the metalworking process. This process is characterized by a high degree of deformation and preferential orientation of the metal grains (Murty and Charit 2006).

### Electrochemistry in SBF

**Fig. 4:**
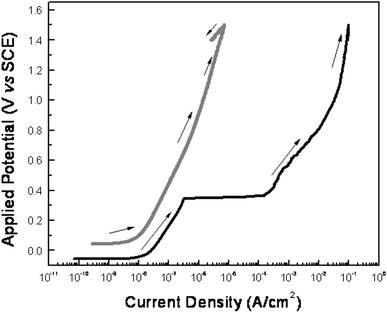
Potentiodynamic polarization curves of zirconium cylinders after 24 h of immersion in SBF solution. (*black line*) control, (*grey line*) anodised

Electrochemical tests were carried out to analyse the protective behaviour and the oxide-induced formation. Anodic polarization curves of anodised and control zirconium samples, after 24-h immersion in SBF solution, are shown in Fig. 4. The corrosion potential (Ecorr) of the control moves in the positive direction compared to the anodised condition, evidencing a nobel surface for the anodic film (Alves et al. 2009; Karthega et al. 2007). Moreover, rupture of the passive film during polarization was observed for the control zirconium samples, whereas no rupture of the anodic film was evidenced in the anodised condition where the material remains in the passive state during the polarization test in the domain of the assayed potentials in this study.

**Fig. 5:**
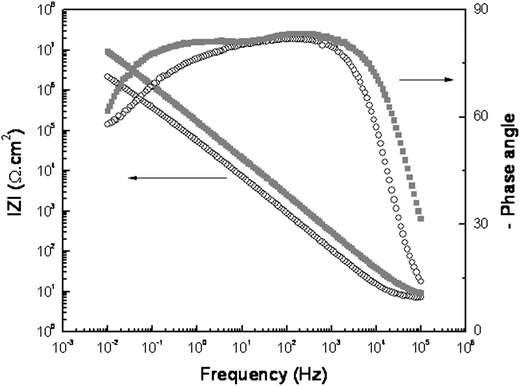
Bode plot of zirconium cylinders after 24 h of immersion in SBF solution. (*open circle*) control, (*filled square*) anodised

Figure 5 shows Bode plots (EIS essays) of control and anodised conditions after 24 h of immersion in SBF. An increase in total impedance of the system for the anodised condition is observed, together with a shift to higher frequencies in the high-frequency domain of the phase angle vs frequency plot, raising the angle close to 90º. Both characteristics of the EIS response can be related to an increase of the barrier effect of the anodic films compared to the native zirconium oxide, which is in agreement with the decrease in current density observed in the anodic polarization curves.

### Histology

#### Clinical observations

The animals recovered well after the surgery and neither signs of infection nor inflammation was noted upon clinical examination during the duration of the experiment.

#### Histological analysis and histomorphometry

**Fig. 6:**
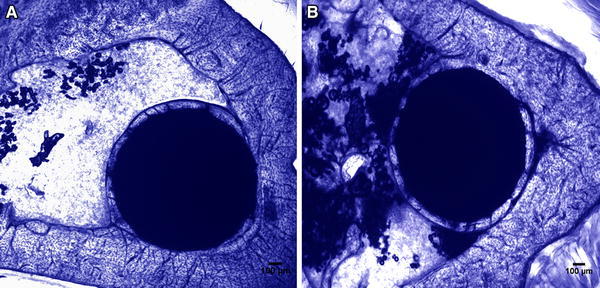
Histology showing bone–implant interface in rat tibia cross section, 63 days after the implantation. **a** New bone formation around control implant. **b** New bone formation around anodised implant (staining: toluidine blue, original magnification 4×)

Figure 6 shows optical microscopy images of toluidine blue-stained section of the anodised implant sample after 63 days of implantation in a rat tibia model. It is possible to distinguish three regions in the histology images, both control and anodised: the remodelling region (where the implant was placed in contact with the endosteal surface of the old cortical bone), the newly formed bone or the novo bone formation zone (with the implant in contact with the marrow medium), and the so-called cortical bone zone. Histological analysis demonstrates that the characteristics of bone tissue integration around control and anodised implants are similar. Lamellar bone is continuous and in close contact in both metal surface implants (bone–implant contact). There is no fibrous tissue between the implant and the bone. No mononuclear cell accumulations (lymphocytes, monocytes) or osteoclast is seen either close to the implant or in the cortical bone.

**Fig. 7:**
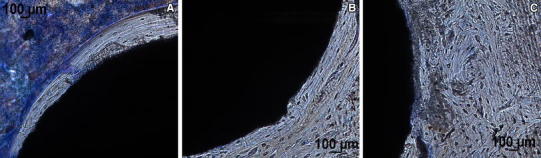
Histology showing bone–implant interface at high magnification in an anodised rat tibia cross section, 63 days after implantation. **a** New bone formation in contact with bone marrow. **b** Remodelation region between the implant and the cortical bone. **c** Cortical bone region (staining: toluidine blue staining, original magnification 20×)

Figure 7 shows the three regions mentioned above at high magnification. It is possible to observe a reduced number of osteocytes embedded in the osteoid matrix in the region corresponding to new bone formation (Fig. 7a) compared to the remodelling region and in the cortical bone zone (Fig. 7b, c).

**Fig. 8:**
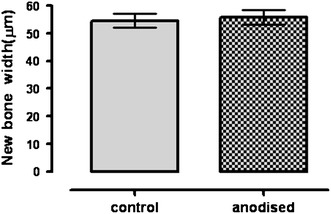
Thickness of new bone layer on the implant surface in control and anodised implants. Data are expressed as mean ± SEM, *n* = 3 tibia/group (by one-way ANOVA and Tukey’s test)

The histomorphometric analysis revealed no significant difference between the newly bone thickness facing the implant between control and anodised samples at 63 days after surgery (Fig. 8).

#### Fluorescence and morphometry analysis

**Fig. 9:**
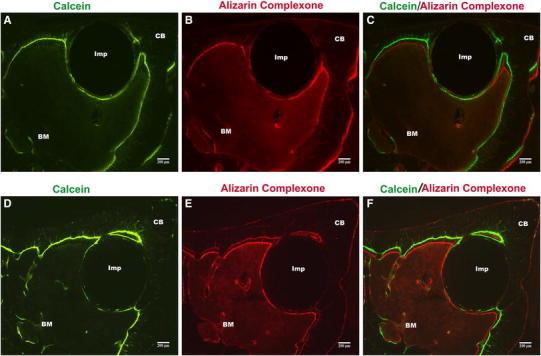
Fluorescent microscopy image of rat tibia cross-sections with control and anodised implant 63 days after implantation. *Green* (Calcein) and *red* (Alizarin Complexone) *lines* are seen in the new bone around the implant (Imp) at 45 or 60 days after implantation, respectively. *BM* bone marrow, *CB* cortical bone (original magnification 40×)

Fluorescent markers calcein and alizarin complexone were detected in control and anodised implants with the same distribution. The staining was found in contact with bone marrow, and no fluorescent signs were found facing the cortical bone. The green line corresponding to calcein was seen close to the metal surface, while the red line was slightly further from the metal surface (Fig. 9a, b in control implants and d, e in anodised one). A continuous formation of new bone showed the double fluorescent labels in both kinds of implants (Fig. 9c, f).

**Fig. 10:**
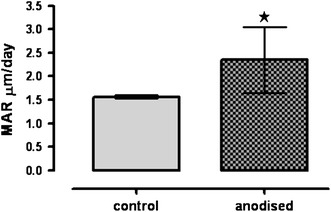
Quantitative analysis of MAR in control and anodised implants. Values are reported as mean ± SEM. *n* = 3 tibia/group (by one-way ANOVA and Tukey’s test). *p < 0.05

A significantly greater MAR was measured in the bone ingrowth and periprosthetic bone around the metallic implant in the anodised implants when compared to the controls (*p* = 0.031) 63 days after surgery (Fig. 10).

## Discussion

Since surface plays a key role in biomaterials performance, its modifications and integrity in bodily fluids due to corrosion phenomena are factors to take into account prior to in vivo testing.

Although chloride ions are known to have detrimental effect on zirconium, polarization curves show an increase in the passive zone for the anodised condition in the absence of a rupture potential, together with a decrease in passive current density when compared to the control. This is indicative of a higher corrosion resistance of the anodised condition, which improves the barrier effect respect to the control. Accordingly, in EIS spectra the increase of total impedance modulus together with a rise in the phase angle plot in the low frequency region, and a shift to higher frequencies that can be related to the increase of the barrier effect for the anodised condition. This fact has already been observed in previous studies conducted on plane zirconium samples where the stability of the oxide is formed and the thickness increase for the anodised samples were demonstrated (Hoerth et al. 2014).

It has been demonstrated that changes in topography and chemistry of the surface greatly affect the tissue response in in vivo assays (Albrektsson et al. 2000). There are many aspects of the early interaction between the implant and the tissue that have not been studied in depth, especially in in vivo models where the complexity of the biological system makes it harder to study the roles.

Orthopaedic and dental implant fixation depends on both bone and bone–implant contact, and bone formation around implant. It is known that gaps at the interface between the implant and the bone increase the risk of failure in implant fixation, which may result in the loss of the piece (Gao et al. 2009). To evaluate the biological response of anodisation treatment in zirconium implants, a rat tibia implantation model was used. This model has been successfully implemented by our group previously (Ballarre et al. 2009). In the literature, different time periods are used when studying implant osseointegration; animals are killed after 2 weeks (Tengvall et al. 2004), 4 weeks (De Ranieri et al. 2005), 6 weeks (Gabet et al. 2008), and 3 months (Gao et al. 2009). In our study, we evaluated implant fixation at 63 days after implantation to assess the static and dynamic process of the bone/implant interface formation. The in vivo results indicate that both control and anodised zirconium implants have continuous laminar bone growth on the surface of zirconium implants in rat tibia. In both cases, it is possible to note a reduced number of osteocytes between the bone marrow and the implant when they are compared with the osteocytes in the remodelling region facing the endosteum and in the cortical bone region. Osteoclasts could not be found in our light microscopy slices. Interestingly, it is possible to find remodelling events extending quite a bit away (100–500 µm) into the cortical bone (Mavrogenis et al. 2009). In the three regions mentioned above in contact with the implant, it is possible to see the osteocytes encased in their lacunae, receiving nutrition and biomechanical signals through the lacuna canalicular system. This suggests that the new bone formed around the metallic implant has the necessary structure to be functional. New bone thickness did not show any significant difference between the control and anodised implants 63 days after implantation. This result is in line with that of Jaatinen et al. (2011) who found that new bone thickness and new bone implant contact were significantly increased 4 weeks after implantation in amorphous diamond (AD)-coated titanium implants. Nevertheless, no changes could be found in both parameters 12 weeks after implantation in rat femur. These results are in line with the results found by Guglielmonti et al. (1999) suggesting that the superficial modification induces a change in bone formation during the acute early phase after surgery, and that at 12 weeks no differences can be found between control and modified implants due to slower bone remodelling after the acute phase. It has been suggested that both the percentage of the implant surface covered by bone and bone thickness may improve osseointegration and anchoring of implants, and it was characterized by mechanical testing. Fluorescent microscopy of the sequential fluorochrome labels revealed the dynamics of bone formation in different periods of implantation (Huang et al. 2009). At 63 days after implantation, the two fluorescent labels calcein and alizarin complexone could be observed with similar intensity. Alizarin complexone was injected after the inoculation of calcein; therefore, alizarin-labelled bone was associated to newly bone deposition. Bone apposition mineralization rate (MAR), based on fluorochrome analysis, was different between the control and anodised conditions. The MAR of bone ingrowth and periprosthetic bone was faster on anodised zirconium implants when compared with control, 63 days after implantation. Interestingly, fluorochrome labelling indicates that the bone extending away from the anodised implant forms at a rate about 25 % faster than that in control implants in the same direction. These results suggest that contact osteogenesis would take place in the region facing the bone marrow. Our observations show that the bone growth rate on the surface of zirconium implant in rat tibia was enhanced by modifying the surface, even 63 days after surgery, suggesting that the anodisation process at 30 V stimulates and assists to the osseointegration process. Osteogenic cells from the endosteum and osteoprogenitors cells from bone marrow could form the new bone close to the control and anodised implant surface all around the implants. This observation is in line with the results found in the histological and fluorescent assays. An important limitation of this study is that only one time point was studied. Further studies are necessary to investigate the early events of bone formation around the metallic surface implants upon the implant is placed. It has been suggested by mechanical testing that bone thickness may improve osseointegration and anchoring of implants (Branemark 1983; Lan et al. 2007; Xiao et al. 2011). In this context we think that biomechanical tests, such as pull-out test, would provide more information about the stability of the interface between the implant surface and the new bone.

## Conclusions

The results of the present study show that surface modification of zirconium implants by anodisation treatment at 30 V can stimulate the implant fixation in a rat model. Within the limitations of this preliminary study, anodised superficial treatment demonstrated a significant increase in bone mineral apposition even 63 days after implantation. This finding is accompanied with a homogenous and compact implant–contact interface all around the implant surface. Based on this study, zirconium should be further investigated as a material for permanent implants, exploring the biochemical and molecular events that take place when the implant is placed.
